# Supplementary material for: Genome-wide identification, characterization and gene expression of BES1 transcription factor family in grapevine (Vitis vinifera L.)
Source: Sci Rep. 2023 Jan 5;13:240. doi: 10.1038/s41598-022-24407-y (PMC9816167; doi:10.1038/s41598-022-24407-y)
Supplement: Supplementary file 3 — Supplementary Information. [file 41598_2022_24407_MOESM3_ESM.zip › Vvi_Atr/Vitis_vinifera.PN40024.v4.dna_sm.toplevel.fa.vs.Amborella_trichopoda.AMTR1.0.dna_sm.toplevel.fa.html/Atr-AmTr_v1.0_scaffold00181.html]

|  |  |  |  |  |  |  |  |  |  |  |  |  |  |
| --- | --- | --- | --- | --- | --- | --- | --- | --- | --- | --- | --- | --- | --- |
| Duplication depth | Reference chromosome | Collinear blocks | | | | | | | | | | | |
| 0 | Atr-ERN03027 |  |  |  |  |  |  |  |  |  |  |  |  |
| 0 | Atr-ERN03028 |  |  |  |  |  |  |  |  |  |  |  |  |
| 0 | Atr-ERN03029 |  |  |  |  |  |  |  |  |  |  |  |  |
| 0 | Atr-ERN03030 |  |  |  |  |  |  |  |  |  |  |  |  |
| 0 | Atr-ERN03031 |  |  |  |  |  |  |  |  |  |  |  |  |
| 0 | Atr-ERN03032 |  |  |  |  |  |  |  |  |  |  |  |  |
| 0 | Atr-ERN03033 |  |  |  |  |  |  |  |  |  |  |  |  |
| 0 | Atr-ERN03034 |  |  |  |  |  |  |  |  |  |  |  |  |
| 0 | Atr-ERN03035 |  |  |  |  |  |  |  |  |  |  |  |  |
| 0 | Atr-ERN03036 |  |  |  |  |  |  |  |  |  |  |  |  |
| 0 | Atr-ERN03037 |  |  |  |  |  |  |  |  |  |  |  |  |
| 0 | Atr-ERN03038 |  |  |  |  |  |  |  |  |  |  |  |  |
| 0 | Atr-ERN03039 |  |  |  |  |  |  |  |  |  |  |  |  |
| 0 | Atr-ERN03040 |  |  |  |  |  |  |  |  |  |  |  |  |
| 0 | Atr-ERN03041 |  |  |  |  |  |  |  |  |  |  |  |  |
| 0 | Atr-ERN03042 |  |  |  |  |  |  |  |  |  |  |  |  |
| 0 | Atr-ERN03043 |  |  |  |  |  |  |  |  |  |  |  |  |
| 0 | Atr-ERN03044 |  |  |  |  |  |  |  |  |  |  |  |  |
| 0 | Atr-ERN03045 |  |  |  |  |  |  |  |  |  |  |  |  |
| 0 | Atr-ERN03046 |  |  |  |  |  |  |  |  |  |  |  |  |
| 0 | Atr-ERN03047 |  |  |  |  |  |  |  |  |  |  |  |  |
| 0 | Atr-ERN03048 |  |  |  |  |  |  |  |  |  |  |  |  |
| 0 | Atr-ERN03049 |  |  |  |  |  |  |  |  |  |  |  |  |
| 0 | Atr-ERN03050 |  |  |  |  |  |  |  |  |  |  |  |  |
| 0 | Atr-ERN03051 |  |  |  |  |  |  |  |  |  |  |  |  |
| 0 | Atr-ERN03052 |  |  |  |  |  |  |  |  |  |  |  |  |
| 0 | Atr-ERN03053 |  |  |  |  |  |  |  |  |  |  |  |  |
| 0 | Atr-ERN03054 |  |  |  |  |  |  |  |  |  |  |  |  |
| 0 | Atr-ERN03055 |  |  |  |  |  |  |  |  |  |  |  |  |
| 0 | Atr-ERN03056 |  |  |  |  |  |  |  |  |  |  |  |  |
| 0 | Atr-ERN03057 |  |  |  |  |  |  |  |  |  |  |  |  |
| 0 | Atr-ERN03058 |  |  |  |  |  |  |  |  |  |  |  |  |
| 0 | Atr-ERN03059 |  |  |  |  |  |  |  |  |  |  |  |  |
| 0 | Atr-ERN03060 |  |  |  |  |  |  |  |  |  |  |  |  |
| 0 | Atr-ERN03061 |  |  |  |  |  |  |  |  |  |  |  |  |
| 0 | Atr-ERN03062 |  |  |  |  |  |  |  |  |  |  |  |  |
| 0 | Atr-ERN03063 |  |  |  |  |  |  |  |  |  |  |  |  |
